# Hollow-core PCF for terahertz sensing: A new approach for ethanol and benzene detection

**DOI:** 10.1371/journal.pone.0320805

**Published:** 2025-03-28

**Authors:** Md. Golam Sadeque, Suchana Aktar Tithi, Md. Safiul Islam, A. H. M. Iftekharul Ferdous, Diponkar Kundu, Md. Galib Hasan, Md. Zakirul Islam Sarker

**Affiliations:** 1 Department of Electrical and Electronic Engineering, Pabna University of Science and Technology, Pabna, Bangladesh; 2 Department of Electrical and Electronic Engineering, Khulna University of Engineering and Technology, Khulna, Bangladesh; Parul University, INDIA

## Abstract

Terahertz (THz) spectroscopy is becoming a powerful technique for non-destructive, label-free chemical sensing with applications ranging from medicinal research to security screening. Enhancing THz spectroscopy’s sensitivity and selectivity is crucial to maximizing its potential. In this work, we offer a novel optical fiber design, square shape core PCF that is tailored to exploit improved optical features at exterior in the THz region. This analysis suggests that a square shape and three layers with square air apertures for the cladding and core would be ideal. The mathematical analysis is carried out at THz wave dissemination utilizing FEM and boundaries circumstance of the Perfectly Matched Layer. Using the simulation method, the constructed square PCF sensor achieves very high relative sensitivity (94.45%, 94.80%) at 2 THz for two compounds: ethanol (n = 1.354), and benzene (n = 1.36). On the other hand, the low confinement loss (CL) values for the same two compounds at 2 THz are 1.17 ×  10^−05^ dB/m, and 1.32 ×  1 0^−05^ dB/m, in that order. We also looked at the potential applications of this special fiber in a variety of fields, including environmental monitoring, chemical sensing, and biomedical diagnostics. The square PCF with square core has hitherto unexplored opportunities for the development of extremely selective and sensitive THz spectroscopic devices with important social consequences in domain of THz perception of chemicals.

## Introduction

PCF is substantial development in optical transmission technology. Because of its distinctive form and regular shifting RI, PCF offers tight control over light propagation. The fiber’s unique composition allows for previously unimaginable variations in flexibility. It can also use shifts, micro structures, and other techniques to enclose photons in its courses. Its latter features transformed several companies, such as healthcare as well as telecommunications. Remarkable flexibility of PC Fiber with regard to light control propels developments in robust information transfer as well as broad spectrum of detector technologies [1,2]. Different time different scholars utilized fiber successfully [3,4]. M.A Eid et al. also using optical fiber on different application [5–7]. This detection is still leading the way of detection technologies this time because it uses unique characteristics to monitor large variety of substances very rapidly as well as precisely alongside least visual data. Fellows measure the amount of heat exertion, elasticity, and even fluctuations inside RI with incredibly high accuracy with utilizing way light engages through its surroundings. This sensors utilize an effective patterning arrangement of PC Fiber, but these also has benefits of small size, portable, unable to withstand radiation disruption, additionally able to work amid challenging circumstances. PCFs have several benefits, including high sensitivity and design flexibility, but they also have drawbacks. Fabrication is complicated and expensive because precise air hole or other structure layouts require advanced manufacturing techniques that are hard to scale for mass production. PCF performance stability may also be affected by external factors including temperature variations and mechanical stress. Hollow-core PCFs, while effective for some sensing applications, can have higher confinement losses and be more susceptible to air hole pollutants, which can impair sensor accuracy over time. PCF integration into current systems may require specialised connectors and equipment, increasing deployment complexity and expense. These limitations emphasize the need for continued research to improve PCF durability, scalability, and practical integration, combining promise potential with real-world limits. Those measurement is helpful tools for many different industries, including aerospace, monitoring the environment, medical care, and construction assessments, because of their high degree of adaptability and versatility. Its flexible attribute transcends monitoring and encourages creativity [8,9]. PCF may be categorized into a trio dependent on the architecture of core. PCFs with porous, solid, and hollow cores are available. Every group can be implemented at the domains for contact in order to guarantee higher security and speedier information transfer. The robust center of The PCF is not appropriate for sensing purposes [10]. The responsiveness of the detector can be improved with more core area, but it is important to recognize that PCF manufacture is a difficult process. A sizable core region is made available for signal transmission by the hollow-core PCF [11,12]. A variety of THz wave routes were proposed by researchers, including insulating, parallel-plate, metal-based, and general fiber wave lines. For direct THz wave applications in sensing and telecommunication from the source to the destination [13]. However, because they absorbed less energy than traditional waveguides, PCFs in the THz area provided better characteristics for signal propagation among them [14]. The term “terahertz spectrum” refers to electromagnetic (EM) waves that have frequencies between 0.1 and 10 THz [15,16].Terahertz frequencies are situated among observable lights and the radio wave FRs region in the electromagnetic spectrum [17]. Gamma radiates and X- radiates are two classes of greater-frequency EM waves which have the ability to propagate outside of the confines of space over long distances, but they also have a tendency to Ionized alloys as well as living biomaterials. Consequently, high-frequency electromagnetic radiation has the potential to cause a multitude of lethal diseases, affecting both humans and animals. However, because terahertz domain lies as opposed to non-ionizing electromagnetic signals, it possible effectively employed in a wide range of medicine and biology [18,19].To transport THz waves from beginning to end, fellows presented range of THz varieties of waveguides, Included in this category are metals waveguides, Bragg fibres, parallel-plate waveguides, and insulating waveguides [20–22]. Over the past many years, number of fellows has paired distinct PCF types with varied sensing samples in an effort to extreme accuracy employing the THz band. Hossain et al. [23]. “Gas detection with low refractive index by a surface plasmon resonance fiber device” was first described by T. Allsop et al [24]. “Examining and analyzing liquid crystal coated photonic crystal fiber interferometers” was the recommendation made by Ginu et al [25].

Various substances can be detected using PCF. For instance, Singh and Kaur [26] detect blood components with a maximal RS of 66.47% and a CL of 1.19 × 10^−08^ dB/m. The RS of 85.8% and CL of 1.62 × 10^−09^ dB/m were achieved by Islam et al. [27] when sensing toxic compounds. In, 2024, With an RS of 91.76% and a CL of 3.50 × 10^−10^ dB/m, Kundu et al. [28] detect fuels. In the same year, Noor et al. [29] demonstrated the ability to identify milk from a variety of animal species with an RS of 88.8% and a CL of 2.04 × 10^−04^ dB/m. Two other terahertz-range PCF detectors were introduced in 2015 by Haxha and Ademgil [30] and Morshed et al. [31]. In 2023, the idea of THz band petrol sensing was also presented by Ferdous et al. [28]. Shakya and Singh developed a biosensor in 2022 and a penta core PC-SPR RI sensor in 2023 [32,33] used different methodologies. Using small elliptical-shaped air holes, the scientist was can obtain highest RS of 49.17% and obtained birefringence values for ethanol,benzene, and water are 0.001513, 0.001514, and 0.001474, respectively. In 2018, a terahertz hollow-core PCF detector was introduced, with Zeonex serving as the fiber basis [34]. There has been a proposal for another THz sensor Kagome-styled exterior siding [35]. Ferdous presented heptagonal air hole circular core in 2021 [36] for terahertz spectrum fuel adulteration detection. The suggested structure likewise has maximum RS of 96.87%and high CL of 8.10355 × 10^−9^ dB/cm [37]. Several PCF-based terahertz detector variations was discussed as well as examined in 2021 [38] in order to ascertain existence of different narcotics, hazardous substances, and blood substances. “A simple PCF-based sensor design with enhanced birefringence and high sensitivity for measuring sulphur dioxide gas” is carried out by Asma et al. [39]. “Using THz spectroscopy to reveal new information about oil goods: a combined RI rectangular core PCF viewpoint identifying,” was the recommendation made by Zannat et al. [40]. “Terahertz spectrum petrochemical sensing: a photonic crystal fiber refractive index hybrid structure approach” was presented by Noor and colleagues [41].

PCF can be used to implement detectors in chemical sensing applications which is extremely tender to shifts in RI of nearby media. This is capable of accomplished by applying detecting substance which engages with target analytes on fiber’s outside. The transmission spectrum of the optic transitions as a result of analyte engagement with detecting material, that changes refractive index of roughly in the middle of fibre. To determine the analyte’s concentration, this shift can be quantified. In recent times, the accuracy of ethanol detection has gained significance for a range of uses, such as industrial, medicinal, and food preservation. Ethanol can be found in drinking alcohol, spirits, and grain alcohol. Ethanol finds application in beverages, fuels, antifungal hand sanitizing gels and use in industry. It is necessary to guarantee that ethanol content is in a precise ratio. Determining the precise concentration of ethanol is essential to minimize the adverse effects.

Benzene is a well-known carcinogen that can cause blood issues including leukemia. Further more, it may affect the brain and spinal cord at high exposure levels, leading to unconsciousness, nausea, and vomiting. Benzene is one volatile organic compound (VOC) that could contribute to air pollution. It is also a component of gasoline and can contaminate the environment and water ways if consumed or leaked. Ethanol is generally considered safe for humans to consume when used in moderation. However, excessive binge drinking can lead to alcohol contamination, liver inflammation, and reliance.

In this work, a novel square-cored square-type PCF for terahertz-based chemical sensing is suggested and examined quantitatively. This design’s produced square PCF sensor shows exceptional relative sensitivity (94.80%, and 94.45%) at 2 THz optimal frequency for two different compounds: benzene, and ethanol. On the other hand, at 2 THz, the NA of ethanol, and benzene are, with very little loss, 0.312, and 0.313 for the same two compounds. This proposed PCF’s square-core and each its round air openings make it easy to assemble using recently established manufacturing techniques. The fuel business may find great use for this highly susceptible square-shaped core-based PCF that will be implemented soon.

## Methodology

**Fig 1:**
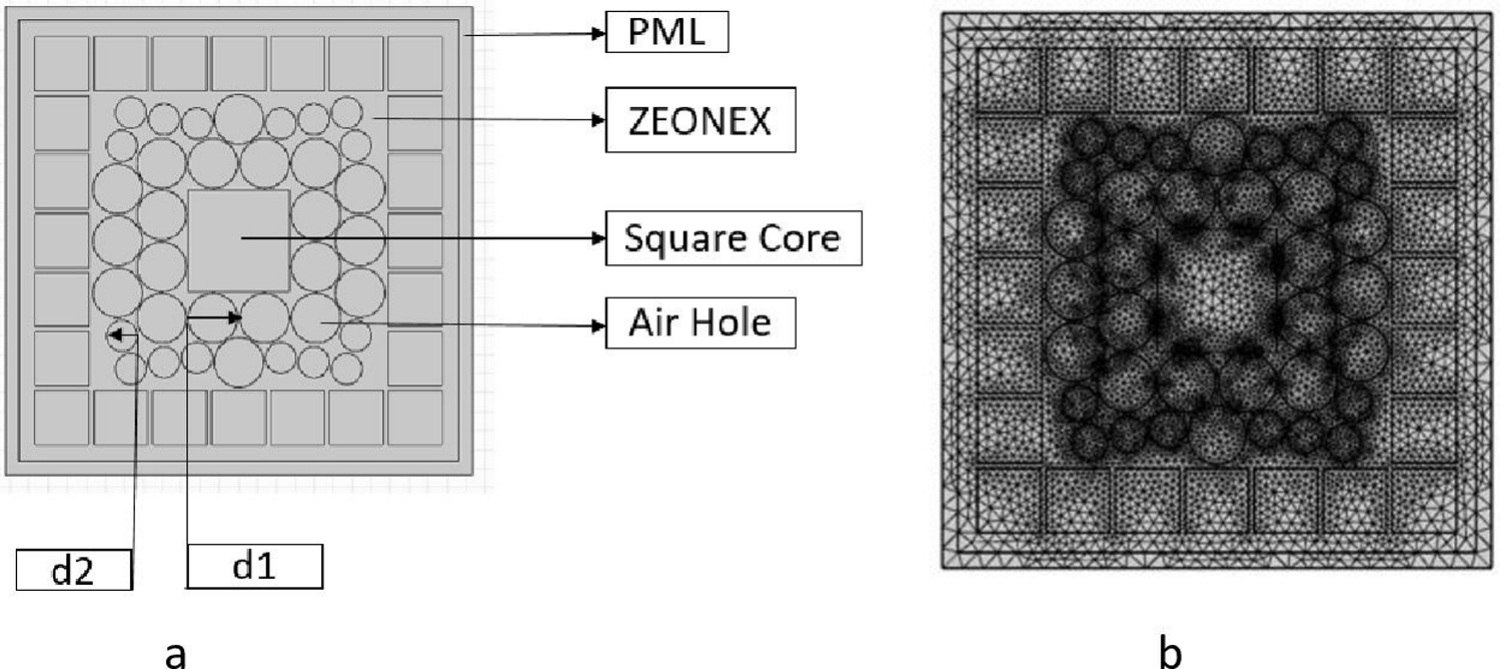
(a) Square-core prototype of the proposed PCF shown schematically (b) An illustration of a square core PCF mesh shown schematically.

The simulations were conducted using COMSOL Multiphysics 6.2 in this study. This software offers a flexible platform for modeling the electromagnetic behavior of PCFs, enabling the precise control of design parameters such as core geometry, cladding structure, and material properties. We were able to optimize the performance of the PCF sensor in the terahertz range by analyzing light propagation characteristics, including confinement loss, relative sensitivity, and effective area, using the finite element method (FEM) within COMSOL. The findings of this study were substantiated by the detailed analysis and validation of the sensor’s design that COMSOL’s robust simulation capabilities facilitated. Central region of proposed framework is shaped of square and packed full of two distinct kinds of chemicals: benzene (RI = 1.36) and ethanol (RI = 1.354) [42]. According to Fig 1a, This design starts with a core that has air openings all around it. Cladding region be mixed-use design made up of numerous square and circular air gaps configure in predetermined design on cladding layer. The square core was chosen for this PCF design because of its capacity to improve the performance of THz sensing applications, particularly for the detection of benzene and ethanol. The square geometry provides numerous benefits, such as enhanced light-matter interaction and polarisation stability, in contrast to circular or hexagonal cores. The corners of the square core contribute to the increased surface area, which in turn enhances the interaction between the analyte and the guided light, thereby increasing sensitivity. This geometry also naturally supports two orthogonal polarisation modes, which is essential for accurate THz sensing. This enhances polarisation stability and reduces noise. Furthermore, the square core enhances field confinement and facilitates a more consistent field distribution, which leads to reduced confinement losses. This design flexibility allowed for the optimal calibration of the PCF’s parameters, thereby maximising its sensitivity to the target analytes in the THz spectrum.

Cladding zone consists of one layer of square-shaped cladding encompassed by massive quantity of air apertures arranged in hybrid structure, as well as circular-air gaps accompanied by various dimensions on superior and inferior aspects of core. We use an air-filling fraction (AFF) of 0.985 to maximize confinement of and simplify manufacturing process. The AFF is the ratio of M to P, in which M denotes dimensions of air openings in cladding region and P approximates their distances between the cells. To achieve good relative sensitivity and minimal CLs, ZEONEX (RI = 1.53) has been utilized as the PCFs sole component. There is a square form for the PML Layer also. PML is utilized to prevent electromagnetic radiation from entering the structure from the outside. The core dimension of a square is 0.7 × p, and the cladding is half of d. First inner ring’s round air holes have a diameter of d1. The structure’s succeeding rings are likewise composed of circular apertures a diameter of d2, where d2 = 16 × d1 and “p” is a variable that varies from 240 to 310 um. A Perfectly Matched Layer (PML) with a thickness of 0.2 times the pitch (p) was implemented at the boundary. This PML configuration facilitates the absorption of outgoing waves and reduces reflections, hence enhancing the precision of propagation characteristic analysis.

A triangle mesh serves as a representation of the design’s whole topology. The element count is 18,738, with triangles totaling 18,738, edge elements 2,370, and vertex elements 252. Element quality fraction is 0.007309, area of mesh is 1.508, and element quality minimum is 0.4696, average is 0.8158, as well as vertex count of mesh is 9,424. To understand sensing properties like sensitivity, CL, NA, EML, and effective area,these elements are essential. Triangular mesh, which is believed to record full object’s layout is shown in Fig 1b. As the detecting analyte, one of two compounds benzene or ethanol is employed. The modeling process then proceeds at several operation stages, spanning from 1.2 to 2.4 THz. Mode matching affects light coupling efficiency between fibre sections or optical components and sensor accuracy, making it a possible problem for the proposed PCF sensor. If the PCF’s guided mode profile doesn’t match other fibres or devices, interface losses may limit sensitivity and cause inconsistent results. PCFs with unusual core geometries, where mode shape might differ greatly from single-mode fibres, are especially affected by this issue. Optimising core size, shape, and refractive index contrast can increase mode compatibility. Mode adapters or tapering portions can improve mode matching, boost light transfer, and reduce signal loss. Optimising these features improves the sensor’s accuracy and reliability, ensuring consistent performance across configurations and applications. The high relative sensitivity for ethanol and benzene detection in this specific hollow-core PCF design is influenced by a number of critical factors. Initially, the square core geometry increases the coupling between the THz wave and the target gases by providing additional surface area at the corners, thereby enhancing the light-analyze interaction. The photonic band gap effect is further enhanced by the meticulously optimized configuration of the triangular air holes in the cladding, which minimizes propagation losses and increases light confinement. This, in turn, contributes to stronger field localization in the core. Furthermore, the specific material selection, Zeonex, provides temperature compensation, guaranteeing consistent sensor performance in a variety of environmental conditions. The efficient propagation of light is facilitated by the low confinement loss (CL) at 2 THz, which maintains the signal’s intensity for precise refractive index measurements. Lastly, the sensor’s sensitivity in the terahertz region is optimized by the precise tuning of the PCF parameters, including the pitch and air hole diameter, which assures a high level of responsiveness to the refractive index changes caused by ethanol and benzene. The square-core PCF sensor has substantial potential in the field of biomedical diagnostics, particularly in the detection of biomolecules or disease markers at THz frequencies. The distinctive square core geometry enables the detection of subtle changes in the refractive index that are induced by biological samples, such as proteins, DNA, or cancerous cells. This interaction between light and matter is essential. The sensor’s high sensitivity enables the precise detection of these bio molecular changes, facilitating the early diagnosis of conditions such as cancer, diabetes, or infectious diseases. The sensor is non-invasive due to its compact design and capacity to operate in the THz region, making it a promising alternative to conventional methods such as PCR or biopsy, which can be time-consuming or necessitate sample extraction. Additionally, the Zeonex material’s temperature compensation guarantees consistent performance in the diverse environments that are characteristic of biomedical applications, thereby improving reproducibility and reliability. This square-core PCF sensor is an appealing instrument for real-time monitoring and point-of-care diagnostics in clinical settings due to its minimal sample preparation, high sensitivity, and portability. Increased sensor robustness to external conditions like temperature and humidity is essential for stability and accuracy in practical applications. Temperature variations can change the refractive indices of the PCF’s core and cladding materials, affecting sensitivity and measurement errors. Temperature-compensating compounds like Zeonex or low-thermal-sensitivity polymers could stabilise the sensor’s performance throughout a variety of temperatures. Moisture in the air holes of hollow-core PCFs can impede light transmission and increase signal loss, therefore humidity control is crucial. Protective coatings or hydrophobic surface treatments can reduce PCF moisture absorption. The sensor can also be protected from environmental changes by sealing or climate-controlling it. These protective measures improve the sensor’s accuracy and reliability in different environments, making it more robust for real-world applications.

**Fig 2:**
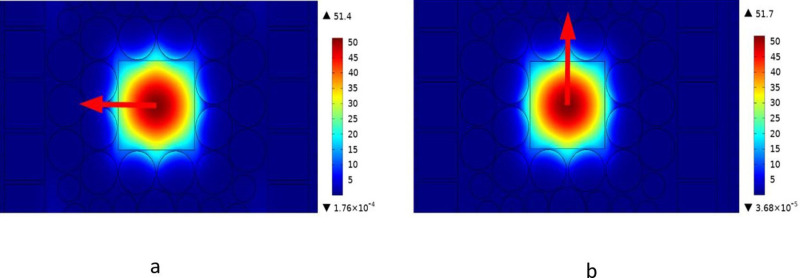
Field distributions for x- and y-pol modes at f =  2 THz, both vertically and horizontally.

Fig 2 shows the directed properties which characterizes photonic concentration of y as well as x modes at the optimal FRs of 2 THz. The substantially enhanced confinement of lite in middle region makes square PCF structure more sensitive to x and y polarization. Evidently, different polarizations cause light passing through fiber to have varying intensity. Consequently, there are significant fluctuations in the fiber’s reactivity with regard to its RS, CL, with other characteristics. Furthermore, the illumination level received by center region from two pol is approximately equal in this configuration. The power distribution is shown in Fig 2 for a particular set of attribute iterations.

A PML border scenario is used in all of the research we did. Permittivity and permeability that are aeolotropic and composite-valued made it possible to identify PML as a material. PML covers the outer rim of the simulation and absorbs all incoming waves. Guided material of detectors among the PM Layer with the outer of core is made of Zeonex. There are many benefits, including minimum material loss, high temperature prejudice, and steady RI while the PML constitutes a tenth of target fiber’s circ. Temperature, humidity, and mechanical vibrations may affect the sensor’s sensitivity and stability. Temperature variations may influence the refractive index of fibre materials or analytes, affecting sensor responsiveness and accuracy. Moisture intrusion into air holes in hollow-core PCFs can impact light propagation and signal loss. Bending or external pressure can distort the fibre structure, affecting light confinement and performance. To make the sensor more resilient, these environmental effects must be studied and protective coatings, temperature-compensating materials, or structural reinforcements added. The sensor would be more reliable in real-world situations and flexible for varied applications in controlled and dynamic environments. The structural configuration and material selection of this proposed PCF sensor design are distinctive, and they have been specifically designed for THz sensing applications. In contrast to conventional PCFs, this design is characterized by a hollow square core that is encased in a Zeonex cladding, which results in reduced dispersion and improved thermal stability in the THz range. Creating a customized bandgap that enhances light confinement and optimizes sensitivity to target analytes, such as ethanol and benzene, the square air pores in the cladding layer are arranged in two distinct diameters (d1 and d2). The boundary reflections are further reduced by employing a Perfectly Matched Layer (PML) boundary with a thickness of 0.2 times the pitch (p). This enables more accurate simulations and improved detection accuracy. This innovative method capitalizes on the PCF structure’s capacity to interact with analytes through refractive index shifts, resulting in substantial enhancements in sensitivity and performance. Consequently, it is well-suited for environmental monitoring and other THz sensing applications.

## Results and analyses

Numerical assessments are conducted on the proposed square PCF using the COMSOL Multiphysics software, and we explain our findings thoroughly to guarantee which signal is confined by liquids or chemicals in order to achieve the desired outcomes. Ethanol and benzene are examples of low RI chemicals or liquid that are used to seal the holes in the middle of the construction. Instead of thesis publications which was first recommended to use in chemical sensors, illustrations or graphic representations were used. To delineate sensor characteristics including sensitivity, effective area, EML, CL, and NA.

Before calculating RS response, it is essential to comprehend characteristics of PCF being employed as detector. RS response of PCF demonstrated its sensing abilities, which were suggested. A PCF’s RS is its ability to adapt its optical properties to modifications in its surroundings, Like changes in pressure or temperature. A number of variables, such as PCF’s architecture, its wavelength, and medium’s characteristics, determine the material’s relative sensitivity to other substances. Because of its huge surface area and limited core size, PCF frequently shows better RS than conventional optical fibers. As per Beer-Lambert’s law, changes in bond between matter and sources of radiation a linear disparity in RS. Here is an equation you may use to find RS value of a sensor[43]:


$$r=\frac{n_{r}}{n_{eff}}\times p\%$$

where *p* is the power fraction, n_r_ is real component of sensing analytes RI; and n_eff_ is guided mode RI.This expression [43] Can be utilized for calculating power percentage.


$$p=\frac{\int_{sample}R_{e}\left(E_{x}H_{y}-E_{y}H_{x}\right)dxdy}{\int_{total}R_{e}\left(E_{x}H_{y}-E_{y}H_{x}\right)dxdy}$$

**Fig 3:**
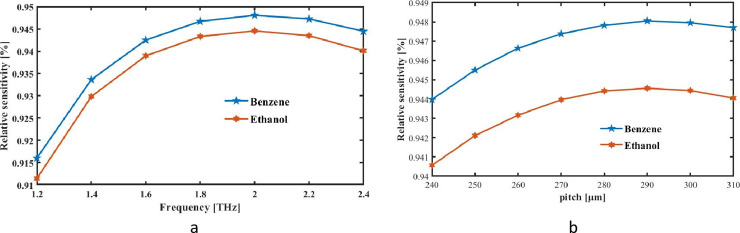
RS is measured as a function of (a) frequency at a constant pitch of 290 μm and (b)pitch when f = 2 THz.

Here, H_x_ and H_y_ represent elements of realm fields of magnetism across x and y pathways, respectively, and E_y_ and E_x_ represent the elements of the electromagnetic field along the same routes. Fig 3a displays the sensitivity curve with respect to frequencies. For proposed PCF-based RI detector, we have run a numerical THz regime study in the 1.2–2.4 THz range. RS curve points progressively and downhill decreases in frequency span as frequency increases. RS of benzene and ethanol reaches its maximum at 2 terahertz and begins to deteriorate at 2.2 THz, as is also shown.

Our proposed square PCF for pitch at THz frequency is shown in Fig 3b along with RS responses. As pitch variable is raised from 240 to 310 μm, the light confinement increases and reaches its highest level at 290 μm. The graphic also shows that benzene have maximum RS, whereas Ethanol has the lowest. At optimal pitch and frequency, the projected square PCF has peak relative sensitivity values of 94.45%, and 94.80% for ethanol, and benzene, respectively. The square air apertures in the cladding were strategically positioned to improve light confinement and reduce propagation losses, thereby enhancing the sensor’s optical properties in the terahertz (THz) region. The photonic band gap effect is more effectively controlled by square-shaped apertures, which restrict the light within the core area, thereby minimizing confinement loss and enhancing field localization in the core. This configuration guarantees that a greater portion of the THz wave interacts with the analytes (ethanol and benzene), thereby increasing the sensitivity. Furthermore, the sensor maintains a high sensitivity of 94.45% and 94.80% across a range of THz frequencies by meticulously optimizing the dimensions and spacing of these square apertures. This is achieved by effectively tailoring the guiding properties to align with the refractive index shifts caused by ethanol and benzene. This design decision guarantees consistent performance for the targeted analytes throughout the THz spectrum by enabling a stable and robust response.

In PCFs, CL refers to the amount of optical power that escapes core of fiber and goes into the cladding area. The outermost material of PCFs is composed of regular rows of air gaps or high-index impurities, which create a photonic band-gap and limit the radiation that can enter the core. It is possible to precisely CL(Lc), by modifying specific structural components.According to [43], actual relation that matches the constraint is presented.


$$L_{c}=\frac{40\pi }{\ln\left(10\right)\lambda }img\left(n_{eff}\right)\times \frac{10^{6}dB}{m}$$

**Fig 4:**
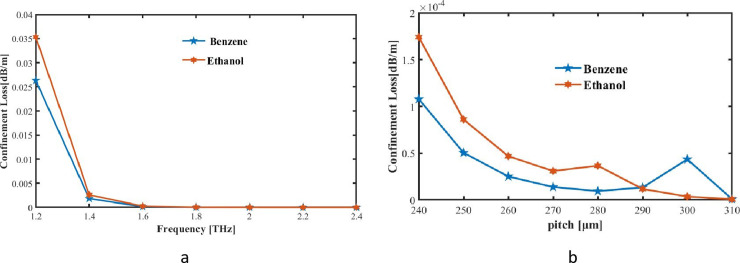
CL at the square PCF we proposed for (a) frequency and (b) pitch as responds.

In this instance, frequency is f and lights speed is c. Fig 4a shows the link between confinement loss and frequency in an ideal setting. Fig 4a illustrates the relationship between CL and frequency, which is unmistakably inverse. CL decreases with frequency, suggesting that higher-frequency modes have better confinement within the optical system and less energy leakage. After 2 THz, CL is nearly nil. This phenomenon highlights the advantages of system at greater wavelengths, including less signal attenuation and more efficient transmission. This group includes two substances: ethanol, and benzene.

Fig 4b shows how the level of CL rapidly falls as the pitches in µm increase for different pitches. It shows rise in pitch and frequency, CL value falls. Under optimum conditions for our proposed square PCF, CL of detector for benzene, and ethanol are 1.32 × 10^−05^ dB/m, and 1.17 × 10^−05^ dB/m, accordingly. The sensor’s overall performance and detection accuracy are considerably improved by the low CL at 2 THz, which ensures that a greater portion of the THz wave energy is effectively contained within the hollow-core PCF. This is essential for the detection of minor refractive index changes caused by the presence of Benzene and Ethanol, as it results in stronger light-analyse interactions. The sensitivity and reliability of the sensor are enhanced by a low CL, which reduces energy leakage into the cladding and maintains the integrity of the THz signal as it propagates through the fibre. The sensor can detect even subtle variations in the refractive index by minimizing losses, resulting in higher detection accuracy. This is especially crucial for the precise and consistent identification of target gases at 2 THz, thereby guaranteeing that the sensor operates at its best across a range of analyte concentrations.

EML in PCF is another important metric that can be caused by both intrinsic and external variables. The specific amount of material loss in a PCF is dictated by its design, manufacturing processes, and operational environment. Scientists and producers strive to minimize material loss by refining fibre’s design, arrangement of materials, and manufacture techniques. To find the exact EML in specific PCF structure, it often takes experimental characterization and measurement techniques tailored to the fiber’s properties. The EML equivalent equation is [43].


$$\alpha_{eff}=\frac{{\left(\frac{\varepsilon_{0}}{\mu_{0}}\right)}^{\frac{1}{2}}\int_{A_{max}}n\alpha_{mat}{\left|E\right|}^{2}dA}{2\int_{ALL}S_{z}dA}$$

**Fig 5:**
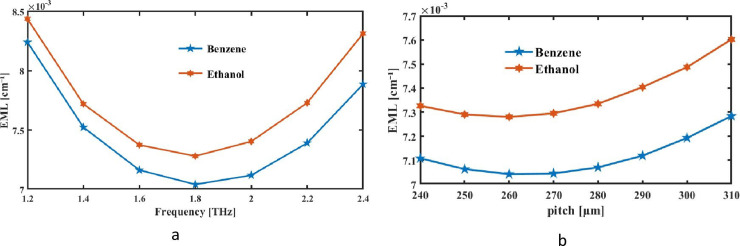
With respect to (a) the signal frequency at fixed pitch of 290 µm and (b) pitch at f = 2 THz, EML of sensing analytes that was supplied is displayed.

Where *E* is electromagnetic fields modality and *α*_*mat*_ is foreground material’s loss factor. Under ideal conditions, Fig 5a and b illustrate how the EML fluctuates for various THz frequencies and pitches based on construction and operating conditions.

The decreasing properties of loss across larger core is shown in the above figures. Zeonex’s wider core can absorb less energy since increased illumination can navigate it with reduced impediments. Higher frequency radiation stays securely contained throughout core, as already demonstrated. Consequently, the EML of proposed device decreases with rising frequency and pitch, as shown in Fig 5a and b.

Fig 5a show frequency increases and the EML of suggested sensors diminishes. The *α*_*mat*_ parameter is close to 0.2 cm^−1^ up to the frequency of 2 THz and then its gets higher. Fig 5b shows that the EML is increased as the pitch for fixed core PCF sensors is increase. These figures detail square PCF fiber’s offered effective mode index reactions with terahertz frequency, as well as the pitch of the EML for benzene (0.0071189 cm^−1^) and ethanol (0.0074747 cm^−1^).

An important guiding variable, NA of a sensor determines highest accepting slant of fiber for incoming light. Usually, the effective RI between fiber’s core and the medium is measured to find the NA in PCFs. It is determined what highest angle is that light can efficiently go through the fiber while being steered along core. An incident ray’s NA is sine of highest angle that it can have for all inside reflections in core of a fiber. By applying formula in, we can find the NA of this particular optical detector type [43] as:


$$NA=\frac{1}{\sqrt{1+\frac{\pi A_{eff}f^{2}}{c^{2}}}}\approx \frac{1}{\sqrt{1+\frac{\pi A_{eff}}{\lambda^{2}}}}$$

**Fig 6:**
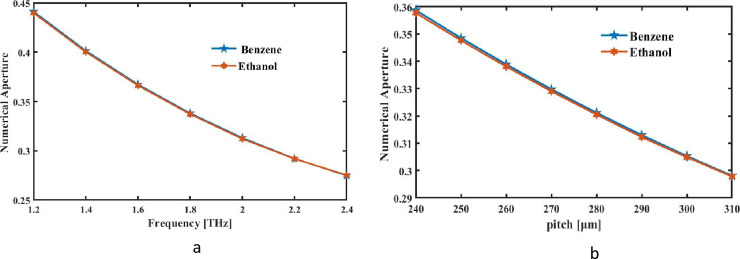
NA of proposed detecting analytes is found with respect to signal bandwidth at constant pitch 290 µm and b pitch at f = 2THz.

Where *A*_*eff*_ stands for operational surface region of detecting analytes with *A*_*eff*_ for appropriate frequency. The numerical aperture using the THz FRs of suggested square sensor for the ideal conditions is shown in Fig 4. With increasing frequency comes a narrower numerical aperture. Fig 6a displays NA relates to changes in frequency. The data show that frequency rise, but NA falls. In Fig 6b, Numerical aperture falls as the pitch value increases. For benzene, and ethanol, the NA values at optimal pitch p = 290 µm and frequency f = 2Terahertz are 0.313, and 0.312, respectively.

Furthermore, an analysis of a detecting fiber’s EA under distinct employed situations is now underway. The entire area that the light stream passes through is measured using EA, which works in a barometer. Area in a fiber’s longitudinal plane where the energy of signal is dispersed is called its effective area. PCFs may be able to store signal by means of conjunction of photonic bandgap and complete introspection due to their unique microstructure. Because PCFs are contained in a way that makes its EA considerable smaller on par with ordinary fiber, they can be used for a variety of purposes. This expression to be utilized to determine EA [44]:


$$A_{eff}=\frac{{\left[\int I\left(r\right)rdr\right]}^{2}}{{\left[\int I^{2}\left(r\right)rdr\right]}^{2}}$$

Where the propagation brought on by the compound being sense’s electromagnetic field is expressed as I(r) = *E*^2^.

**Fig 7:**
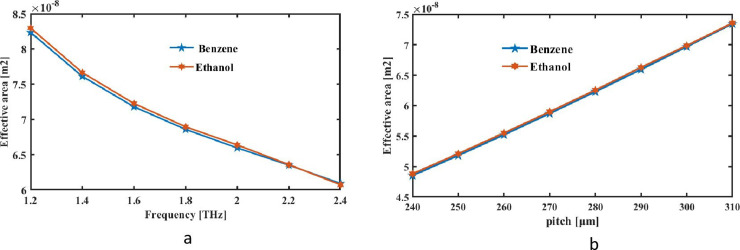
EA is measured as a function of (a) frequency at a constant pitch of 290μm and (b) pitch at a constant f = 2 THz.

Fig 7 chosen sensing analyte’s EA for a constant pitch of 290 µm and a variable pitch of Fig 7a shows expected EA of the square PCF as an estimate of frequency for most suitable settings. It shows how EA of this design decreases with increasing operational frequency. In lattice of PCF, wavelength of light drops with increased light frequency, resulting in reduced geographical time.EA decreases as a result of the photonic crystal structure’s narrower spacing brought about by the shorter spatial period. Fig 7b shows EA responses at proposed sensor for optimal pitch at f = 2THz.

Increasing pitch causes the effective area to expand. A larger pitch essentially creates a wider core region by increasing the apparent area between the air gaps. Bigger core size facilitates greater confinement of photonic strength within core, hence increasing effective area. EA for ethanol, and benzene at the optimal frequency and pitch were 6.728 × 10^−08^ m^2^, and 6.694 × 10^−08^ m^2^, accordingly. PCFs have a bright future with potential for improvements in performance and applications. Research is improving fabrication methods to lower costs and make complicated PCF structures more economical for large-scale production. Advanced polymers and nano-composite coatings may improve PCF thermal and chemical resilience, making them suited for harsher settings. Design innovations like hybrid PCFs with solid and hollow-core areas could modify light-matter interactions and increase sensitivity for specialised sensing applications. Functionalised PCFs with biocompatible coatings may enable precise, non-invasive diagnostic instruments in biomedicine. PCFs will bridge the gap between laboratory research and real-world applications in environmental monitoring, telecommunications, and healthcare as they evolve. Despite the PCF sensor’s high sensitivity, analyte interaction can limit its effectiveness, especially for low concentration detection and fast response times, according to the study. Light-analyte interaction is limited by fibre shape and air hole distribution around the core. Increased core surface area or cladding air hole design and size could improve light confinement and analyte interaction, increasing sensitivity. Additionally, matching the core and cladding materials to the target analytes’ refractive index could boost interaction strength, allowing the sensor to detect even smaller changes. Coatings or functionalized surfaces on fibres could improve selectivity and sensitivity by binding target molecules. These adjustments may increase the PCF’s sensitivity, making it more useful in more applications.

### Comparison with previous works

Chemicals detection is important and in great demand in the industrial and practical sectors. Examples of this include the detecting of water, benzene, ethanol, and other organic compounds. Chemical concentration levels can be determined using only a handful of established procedures. But they are expensive, tedious, and made of mistakes easily. Researchers have so zeroed in on sensors based on PCF as a means of measuring chemical concentrations. Some writers have recently introduced many kinds of PCF-based sensors for ethanol, benzene, and water detection, among other compounds. Results from comparing these works to other published studies and tweaking the ideal parameters for offered square PCF detector operating at 2 THz are displayed in Table 1. At 1.6 terahertz frequency, a kagome-structured PCF-based detector with rectangular organized air openings were suggested in [45] with shown RS of 85.6%, 85.7%, and 85.9%. In [46], a hybrid PCF detector with a CL of 2.53 ×  10^−09^ dB/m and the greatest 89% RS was presented, using solely benzene as an analyte. Sen et al. sense ethanol, benzene and water with RS 82.26% and CL 6.07 × 10^-08^ dB/m in FRs 1 THz [47]. Iqbal et al. detect Ethanol with RS 91.50% and CL is 1.51 × 10^-12^ dB/m in FRs 2 THz [48].

**Table 1:** A study comparing results of earlier published sensors with that of suggested sensor layout.

Citations	PCF’s layout	Analysts	Frequency (THz)	Sensitivity (%)	CL (dB/m)	EML (cm^-1^)
PCF [45]	Kagome-designed with air holes that are constructed in a rectangle	Water	1.6 THz	85.6%	4.5 × 10^-09^	–
		Ethanol		85.7%	1.71 × 10^-09^	–
		Benzene		85.9%	1.02 × 10^-09^	–
PCF [46]	HC PCF alongside rectangular core	Benzene	1.7 THz	89%	1.15 × 10^-09^	0.02
PCF [47]	Hexagon clad as well core	Ethanol	1 THz	81.46%	5.85 × 10^-08^	–
		Benzene		82.26%	6.07 × 10^-08^	
		Water		79.22%	5.84 × 10^-08^	
PCF [48]	Rectangular-based structured	Ethanol	2 THz	91.50%	1.51 × 10^-12^	0.0064
PCF [49]	Square clad alongside octagon core	Water	1.3 THz	90.65%	1.88 × 10^-08^	0.0136
		Ethanol		91.93%	2.00 × 10^-08^	0.0146
		Benzene		93.06%	2.12 × 10^-08^	0.0156
PCF [50]	PCF-based Octagonal Chemical Sensor Design	Ethanol	1 THz	91.25%	5.95 × 10^-08^	0.0131
		Benzene		93.80%	5.94 × 10^-08^	0.0133
This PCF	An square cladding with a square core	Ethanol	2 THz	94.45%	1.17 × 10^-05^	0.0074
		Benzene		94.80%	1.32 × 10^-05^	0.0071

This research shows that a square PCF-based detector can tell the difference between ethanol and benzene as two separate chemicals. Main parts of the sensor’s form are a round air hole and a square cover with a square air hole. The square core made it possible for almost no confinement loss for the two chemicals above, which helped the sensor’s RS go up by 94.80% and 94.45%, accordingly. Assessment with other PCF-based chemical sensors is shown in Table 1.

A thorough literature study shows that the proposed PCF sensor architecture has many benefits over previous structures. Kagome-designed PCFs with rectangular air hole configurations [45], hexagonal and rectangular cores [46,47], and rectangular or square cladding with octagon or rectangular cores [48,49] have been studied. These systems offer good sensing but lack the optimal structure for THz applications. In contrast, the suggested PCF uses a square core and square cladding air holes of different diameters to improve light confinement and THz sensing refractive index sensitivity. This square-clad, square-core structure optimizes mode field distribution and simplifies production, improving sensitivity and confinement loss. This sensor is designed with Zeonex coating for temperature stability, which is important for applications in different environments. These enhancements make the proposed PCF sensor ideal for high-sensitivity target analyte detection, overcoming the limitations of previous works. The Finite Element Method (FEM) and PML boundary conditions were employed to simulate the propagation of THz waves, but they encountered numerous obstacles. Accurately modeling the interaction of THz waves with the intricate structure of the hollow-core PCF, particularly with the square core and air aperture geometry, was one of the primary challenges. In order to prevent interference with the propagation of waves within the fibre, it was essential to ensure that the PML boundaries effectively absorbed outgoing waves without producing reflections. Furthermore, the FEM simulations necessitated fine-tuning the mesh resolution to achieve precise results without computational overburden, as THz waves are highly sensitive to the fiber’s geometry. The precise calibration of the PML layers was another challenge, as it was necessary to prevent numerical artifacts at the boundary from distorting the simulation results. In spite of these obstacles, the simulation parameters were meticulously optimized to enable accurate predictions of the wave behavior and sensor performance in the THz region. In comparison to current THz sensing technologies, the hollow-core PCF sensor that has been proposed provides a number of specific advantages for environmental monitoring. Initially, its extraordinary sensitivity, particularly in the context of detecting gases such as benzene and ethanol, allows for the more precise detection of contaminants at even the lowest concentrations. The square core and triangular air hole configuration improve confinement and enhance light-analyte interaction, enabling more dependable measurements in intricate environmental conditions. Furthermore, the sensor’s capacity to function efficiently in the THz region guarantees that it can identify a wide variety of chemical substances with minimal interference from background noise. The sensor’s stability and reliability in fluctuating environmental temperatures are further enhanced by the use of Zeonex for temperature compensation, a critical factor in outdoor or real-time monitoring. The PCF sensor’s compact and efficient design renders it more suitable for portable and cost-effective deployment in a variety of environmental monitoring applications, including hazardous substance detection and air quality monitoring, in contrast to conventional methods that may necessitate bulky equipment or are restricted by sensitivity.

Lastly, this part talks about how to make a recommended square core sensor. In the past few years, distinct construction PCFs have been developed via a range of methods, such as sol-gel, extrusion, stacking, jacketing, collapsing, and drawing with a standard drawing tower [51]. One of the several process types used in [52] is sol-gel, which allows for a high degree of design control over the core’s size and shape. If the goal is to make the fiber we proposed, then can use this sol-gel to do it. It works for any sort of fiber. Table 1 displays the results of a comparison between the recommended fiber’s key properties and a number of other structural designs discussed in the literature; this will show how this fiber is superior to the ones that have come before. These technologies could potentially enable hybrid core forms and multi-layered cladding structures, which could improve light confinement and sensitivity. These approaches can also combine doped polymers or nano-composite architectures to improve fibre thermal and chemical stability. These novel fabrication methods may make high-performance PCF sensors more affordable and suitable for environmental sensing and biological diagnostics. Experimental validation of the simulation results is an essential stage in verifying the practicality and performance of the square-core PCF sensor. Experimental validation would likely entail the fabrication of the PCF sensor using the optimised simulation parameters and its subsequent testing under controlled conditions with known concentrations of ethanol and benzene in the terahertz region. The accuracy and reliability of the design would be guaranteed by measuring the sensor’s response to these target gases and comparing it to the simulation predictions. This PCF sensor has substantial potential for environmental monitoring in real-world applications, including the detection of hazardous gases and the measurement of air quality in industrial or urban settings. Furthermore, its non-invasive nature and high sensitivity render it appropriate for biomedical diagnostics, particularly for the early detection of diseases through the identification of biomarkers in biological samples. Additionally, the sensor’s portability, stability, and compactness render it an appealing choice for portable devices. This enables point-of-care applications in healthcare settings and offers a promising future for widespread use in both environmental and medical sectors. Practical implementation requires integrating the PCF sensor with terahertz (THz) sources and detectors, but more research is needed to assure smooth operation and optimal performance. THz systems have distinct mode profiles and require high precision to avoid coupling losses, making it difficult to efficiently couple THz waves into and out of the PCF sensor. To maintain signal intensity and sensitivity, the PCF, THz source, and detector must be aligned. Misalignment might lower detection accuracy. Couplering methods like specialised interfaces or tapered fibre ends may increase light transfer. Compact and portable THz sources and detectors make the arrangement more field-friendly. These integration issues can be resolved to improve the sensor’s usage in environmental monitoring and biomedical diagnostics.

## Conclusion

In order to identify ethanol and benzene, an square shape and core chemical sensors is created and modeled in this study using COMSOL Multiphysics. So that it can absorb EM-energy released by the sensor, a PML was built and used. We describe an square PCF that is capable of terahertz waveguide frequency analyte detection. Both the PML boundary condition approach and the FEM method are used to accurately complete all numerical calculations. Outstanding photonics characteristics of offered square PCF sensor include lower confinement losses of 1.17 × 10^−05^ dB/m, and 1.32 × 10^−05^ dB/m for ethanol, and benzene at 2 terahertz frequency range, and better relative sensitivity of 94.45%, and 94.80%. Rather, there are certain drawbacks to this arrangement, including mode matching, compatibility with different materials, restricted analyte interaction, and susceptibility to the environment. Future work can focus on environmental resilience, integrating with THz-sources and sensors, and improving fabrication methods. By resolving these issues and following these avenues of future investigation, domain of THz chemical identification using square-core square-shaped PCF may be significantly ahead of, making it a valuable instrument in a variety of scientific, industrial, and security applications.
